# SAR Image Change Detection via Multiple-Window Processing with Structural Similarity

**DOI:** 10.3390/s21196645

**Published:** 2021-10-06

**Authors:** Minseok Kang, Jaemin Baek

**Affiliations:** 1 Division of Electrical, Electronic, and Control Engineering, Kongju National University, Cheonan 31080, Korea; mskang@kongju.ac.kr; 2 Department of Mechanical Engineering, Gangneung-Wonju National University, Wonju 26403, Korea

**Keywords:** change detection, multiple-window processing, gamma correction, synthetic aperture radar, structural similarity index measure

## Abstract

In this paper, a synthetic aperture radar (SAR) change detection approach is proposed based on a structural similarity index measure (SSIM) and multiple-window processing (MWP). The proposed scheme is performed in two steps: (1) generation of a coherence image based on MWP associated with SSIM and (2) gamma correction (GC) filtering. The proposed method is capable of providing a high-quality coherence image because the MWP operation based on SSIM has high sensitivity to the similarity measure for intensity between two SAR images. By finding an optimum value of order of GC, the proposed method can considerably reduce the effect of speckle noise on the coherence image, while retaining nearly all the information related to changed region involved in the change detection map. Several experimental results are presented to demonstrate the effectiveness of the proposed scheme.

## 1. Introduction

The change detection (CD) technique has been widely applied in the evaluation of monitoring and predicting trends in disasters that have occurred in the investigated area by using synthetic aperture radar (SAR), which can operate day and night and during almost any weather conditions [1,2,3,4]. The generation of a CD map (CDM), a kind of a binary change mask corresponding to difference between two SAR images, is aimed at providing the dynamic evolution of scene changes [5,6,7]. However, SAR images usually suffer from the presence of speckle noise, which leads to the degradation of detection performance of CD approach [8,9,10].

The SAR change detection methods can be divided into two groups: non-coherent (NC)-based CD (NCCD) and coherent-based CD (CCD) approaches. The NCCD techniques the subtraction and ratio operators (e.g., difference image (DI), log-ration difference (LRD), logarithmic mean-based thresholding (LMT) and neighborhood-based ratio approach (NRA)) have been successfully proposed. The DI [11] and LRD [12] are widely used for generation of CDM. The binary classification of changed region between two SAR images is presented in [13] using the LMT approach. The NRA is proposed in [14] considering the gray level and spatial information of neighbor pixels caused by DI. A novel SAR image CD method using saliency extraction and the shearlet transform (SEST) is proposed in [15]. In [16], the authors derive a statistical hypothesis approach based on bivariate gamma distribution (BGD) for wavelength-resolution NCCD. On the other hand, CCD techniques such as cross-correlation (CC) is successfully applied in handling the issue of SAR CD task. The coherence image is calculated by the CC using a small sliding window of pixels across the complex interferogram whose resulting phase information involves ground motion and surface change with unprecedented spatial detail [17]. Compared with the classic CC, the similarity measure operators such as Berger and universal quality index (UQI) [18] can decrease the influence of calibration and radiometric errors and is more suitable for the CD of SAR images. The speckle noise of SAR images is multiplicative noise and the similarity measure operation can not only transform multiplicative noise into additive noise but also compress the value range of pixels in an SAR image. However, lack of coherence caused by several decorrelation factors such as baseline, temporal, and rotational decorrelation has a strong influence on the performance of the accurate CD result.

The main contribution of this paper is to present a novel framework for CDM generation using the multiple-window processing (MWP) associated with the structural similarity index measure (SSIM) [19,20] and gamma correction (GC) [21,22,23]. The proposed method is capable of providing high-quality coherence image since the MWP operation based on SSIM has high sensitivity to the similarity measure for intensity between two SAR images, compared to classical similarity measures [24,25]. Meanwhile, the detection performance of the coherence image is dependent on the order *p* of GC [22]. The proposed method can obtain various degrees of performances of the speckle noise reduction with changes in the order *p* of GC. Thus, we analyzed the proposed method in terms of the image enhancement and the speckle noise reduction, taking the value of *p* into consideration. It achieves a superior quality of CDM by searching the value of *p* such as the global optimal solutions of the optimization problem with the GC technique. In experimental results, several detection quality metrics are employed to quantitatively evaluate the performance of CDM acquired by the proposed method.

The remainder of this paper is organized as follows. Section 2 presents some useful concepts regarding in the MWP associated with SSIM. In Section 3, the optimization task based on gamma correction is explained in detail. In Section 4, the computational complexity of the proposed algorithm is analyzed in mathematical expression. The results of several experiments are presented in Section 5 to testify to the performance of the proposed method. Section 6 discusses the results in perspective of previous studies, pointing out some future research directions. Finally, Section 7 presents our conclusions.

## 2. Analysis of MWP Associated with SSIM

Consider the case where we acquire two SAR images at different times but of the same geographical area on the Earth’s surface [25]. The CD can be performed using the complex CC between the master and the co-registered slave calculated locally over a small, namely 5×5 or 10×10, pixel window in the SAR image pair [3]. However, the CC approach often fails to achieve excellent creation of the CDM due to the limitation of accuracy of similarity measure in presence of several decorrelation factors.

In this paper, we focus on the behavior of the MWP based on SSIM in SAR image, which is capable of outperforming CD ability more than CC in similarity measurement. Because each pixel intensity in image is closely related to neighborhood and represents dynamics of the scene being imaged, the proposed approach can exhibit a simple structure and powerful detection performance. Let a pair of two co-registered SAR images with a size of M×N, where *M* and *N* are the number of pixels in azimuth and slant-range domain, respectively, be $I_{1}=\left\{i_{1}(m,n)\left|1\leq m\leq M,\;1\leq n\leq N\right.\right\}$ and $I_{2}=\left\{i_{2}(m,n)\left|1\leq m\leq M,\;1\leq n\leq N\right.\right\}$, respectively. The coherence image located at (*y*, *x*) is calculated by SSIM using a small sliding window of pixels across both absolute images ($\left|i_{1}(m,n)\right|$ and $\left|i_{2}(m,n)\right|$) and can be expressed [19]:

(1) $$\gamma_{ssim}(y,x,M^{\prime },N^{\prime })=\frac{\left(2\bar{\alpha }\bar{\beta }+c_{1})(2\sigma_{\alpha \beta }+c_{2}\right)}{\left({\bar{\alpha }}^{2}+{\bar{\beta }}^{2}+c_{1})(\sigma_{\alpha }^{2}+\sigma_{\beta }^{2}+c_{2}\right)}$$

where

$$\sigma_{\alpha }=\sqrt{\frac{1}{N^{\prime }M^{\prime }-1}\sum_{n=1}^{N\prime }\sum_{m=1}^{M\prime }{\left(\left|i_{1}(m,n)\right|-\bar{\alpha }\right)}^{2}},\;\;\;\;\;\sigma_{\beta }=\sqrt{\frac{1}{N^{\prime }M^{\prime }-1}\sum_{n=1}^{N\prime }\sum_{m=1}^{M\prime }{\left(\left|i_{2}(m,n)\right|-\bar{\beta }\right)}^{2}},$$

$$\sigma_{\alpha \beta }=\frac{1}{N^{\prime }M^{\prime }-1}\sum_{n=1}^{N\prime }\sum_{m=1}^{M\prime }\left(\left|i_{1}(m,n)\right|-\bar{\alpha })(\left|i_{2}(m,n)\right|-\bar{\beta }\right),$$

$$\bar{\alpha }=\frac{1}{N^{\prime }M^{\prime }}\sum_{n=1}^{N\prime }\sum_{m=1}^{M\prime }\left|i_{1}(m,n)\right|,\;\;\;\;\;\;\;\;\;\;\bar{\beta }=\frac{1}{N^{\prime }M^{\prime }}\sum_{n=1}^{N\prime }\sum_{m=1}^{M\prime }\left|i_{2}(m,n)\right|,$$

$$c_{1}={\left(k_{1}D\right)}^{2},\;\;\;\;\;\;\;\;\;\;\;\;\;\;\;c_{2}={\left(k_{2}D\right)}^{2},\;$$

**Figure 1:**
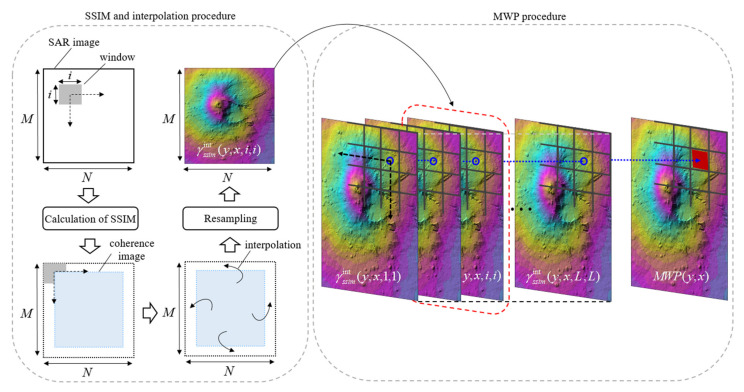
Schematic diagram of the SSIM and MWP procedure.

$M^{\prime }$ and $N^{\prime }$ are the *y*-directional and *x*-directional window widths, respectively. The size of the $M^{\prime }\times N^{\prime }$ image segment is usually much smaller than the size of the M×N image. Note that the local window with a size of $M^{\prime }\times N^{\prime }$ is typically chosen as square in this paper. *D* is the dynamic range of the pixel values. In addition, $k_{i}(i=1,2)$ is constant indicating scaling factor. According to numerous computer simulation results with an exhaustive search procedure for finding the best value of $k_{1}$ and $k_{2}$, it is preferable to optimize the SSIM using the parameters $k_{1}=0.01$ and $k_{2}=0.03$ appropriate for providing the reliable performance within the framework of the proposed method. Structural information is the idea that the pixels have strong inter-dependencies especially when they are spatially close. These dependencies carry important information about the structure of the objects in the visual scene. Thus, it is more preferable to select the SSIM rather than conventional similarity measures such as CC and UQI [24] owing to its ability to measure information loss in terms of image similarity measurement. In the pursuit of similarity detail between two images, the SSIM converges to 1 if both images are identical. The SAR image pair, contaminated by a high frequency random speckle noise, has been filtered using a low-pass filter designed by the windowing of SSIM operation. The low-pass filter will pass the signal through it and suppress the high frequency speckle noises in SAR image. Thus, reliable reduction in the speckle noise can be ensured in the configuration of SSIM operation. Meanwhile, the resultant of SSIM associated with the multiple-window with different size of windows can be added cumulatively to preserve the detail information such as edge of the changed region. As shown in Figure 1, the MWP, obtained from the average of SSIM results for each different window, can be derived as

(2) $$MWP(y,x)=\frac{1}{L}\sum_{i=1}^{L}\gamma_{ssim}^{\mathrm{int}}(y,x,i,i),$$

where $\gamma_{ssim}^{\mathrm{int}}(y,x,i,i)=\mathrm{interp}\left[\gamma_{ssim}(y,x,i,i),\;L,\;L\right]$, *L* is the number of windows at (*y*, *x*). A function of interp returns interpolated values of $\gamma_{ssim}(y,x,i,i)$ at specific query points related to original image size of M×N using interpolation method. After the SSIM transformations were performed at each window size, the interpolation process is applied to one of the SSIM images so that all images are registered at the same pixel location. Then, after the interpolation is used to resample the SSIM image to provide the pixel-level registration, the MWP associated with SSIM is performed to reconstruct high-quality coherence images with the preservation of the detail information such as the edge of the changed region. However, it is inevitable that the SAR images usually suffer from the presence of the speckle noise [26,27]. The coherence image with the inclusion of undesired speckle noise may create challenges in providing reliable detection performance of CDM [28,29]; thus, there is a demand for a speckle noise reduction technique.

## 3. Optimization Task Based on Gamma Correction

Before applying the gamma correction (GC) to reduce the speckle noise, we applied the negative transformation (NT) in advance to reverse the intensity levels of an input image to produce the negative image. The negative image *x* can be expressed as [22]

(3) k=1−MWP(y,x),

where 0≤k≤1. The NT is suitable for enhancing dark detail (low coherence) embedded in white regions (high coherence) of an image, especially when the white regions are dominant in whole image such as coherence image.

**Figure 2:**
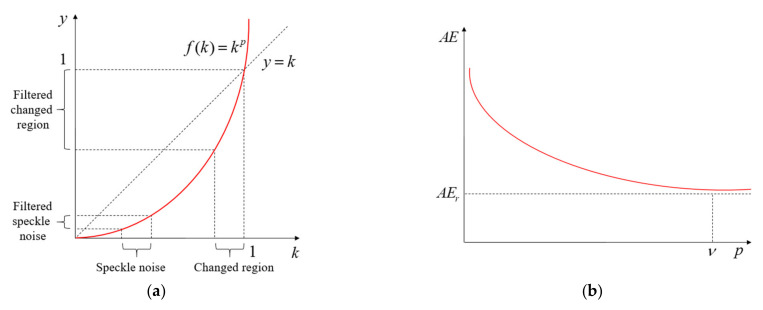
(**a**) Illustration of the GC filtering, (**b**) *AE* versus value of *p*.

The power-law transformation, called GC, can be defined as follows [23]:

(4) $$y=ak^{p},$$

where *k* and *y* are the intensity levels of the pixels in the input and the output CDMs, respectively. *p* is an arbitrary real number. *a* is a positive constant and is selected as 1 in this paper. In GC filtering, the order *p* controls the slope of the transformation function. The higher the value of *p* is, the steeper the transformation curve becomes. The steeper the curve is, the more the corresponding intensities are spread, causing more increase in contrast. Figure 2a demonstrates a plot of *y* with respect to *k* using the order *p* which shows an increasing curve. Note that steeper the transformation curve will be obtained, which will cause large decrease in speckle noise, as expected. Because the intensity of the speckle noise in the CDM is usually smaller than that of the true changed region, the GC filtering leads to a significant reduction in the speckle noise, while retaining nearly all the information related to true changed region contained in the CDM, as shown in Figure 2a. The GC can provide various degrees of performances of both image enhancement and speckle noise reduction with changes in the value of *p*. Thus, the result obtained from the GC can be extremely close in appearance to the noise-free CDM if it is available for the proper selection of *p*. Because the performance of GC has a strong dependence on the value of *p*, the heuristic approach, which suboptimally selects the best solution by optimizing an average energy (*AE*) of CDM, is required to find a proper value of *p*. The GC uses different values of *p* for different images depending on the nature of the respective image according to the *AE* of CDM. Then, the *AE* can be defined as

(5) $$AE(p)=\frac{1}{NM}\sum_{n=1}^{N}\sum_{m=1}^{M}k^{p}(m,n).$$

The *AE* decomposes into two main contributions: $AE_{r}$ corresponding to the changed region and $AE_{s}$ corresponding to the speckle noise as follows:

(6) $$AE(p)=AE_{r}(p)+AE_{s}(p).$$

It is noteworthy that there inevitably exists a certain real number ν which leads to the result that $AE_{s}$ distributed in CDM starts to converge to 0 in the limit as p→ν, that is,

(7) $$\underset{p\to \nu }{\lim}AE_{s}(p)=0.$$

This is because the decreasing rate of $AE_{s}$ is generally larger than that of $AE_{r}$ with the value of *p* increasing as shown in Figure 2a. Thus, this approach results in the removal of the speckle noise in CDM due to $k_{s}^{v}\ll 1$, where $k_{s}$ denotes the NT corresponding to the speckle noise. Therefore, the *AE* has a tendency to converge to $AE_{r}(v)$ close to the noise-free CDM as shown in Figure 2b, and then the following relationship holds true:

(8) $$\underset{p\to \nu }{\lim}AE(p)=AE_{r}(\nu ).$$

Thus, the selection of the *p* parameter at the start of convergence of *AE*, namely p=ν, can reasonably lead to a global optimum in terms of both image enhancement and speckle noise reduction. Then, the reconstruction of desired CDM can be achieved by solving the following optimization problem:

(9) $$\left(P\right):\;\min\;p\;subject\;to\;\frac{d}{dp}AE(p)\geq \sigma ,$$

where σ is small negative threshold value. It is necessary to construct approach to a systematic process in setting for the adaptive threshold selection. According to numerous simulation results with an exhaustive search procedure of *p* parameter against threshold σ, the mathematical expression of the threshold $\sigma ={\left.AE^{\prime }(p)\right|}_{p=1}\times \left[\log L^{2}\right]/100$ is heuristically derived to achieve the global optimum within the SAR NC-CD framework, regardless of the shape and size of changed region and the radar parameters. The initial differential coefficient ${\left.AE^{\prime }(p)\right|}_{p=1}$ is considered as the data-driven parameter to describe the proportion of the changed region in the original image. Finally, the CDM can be created where clusters of pixels with intensity above a specified threshold represent the scattering center locations on the changed region.

**Figure 3:**
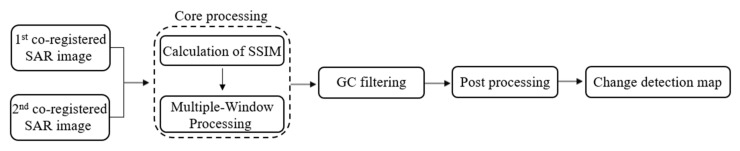
Detailed processing workflow that is proposed in this study.

Additionally, we can consider a median filter with size of 3×3 in the post-processing step to accommodate the residual speckle noise reduction in the CDM. The overall flowchart of the proposed method is shown in Figure 3.

## 4. Analysis of Computational Complexity

The computational complexity of the SAR change detection for the proposed method is analyzed in detail. We establish the computational efficiency of the proposed method with regard to multiplications and divisions. Given *N* (N≥2) SAR images with a size of M×N, the total complexity of SSIM requires $O\left(L\cdot M\cdot N\cdot (N-1)\right)$ operations, where L is the number of windows in the MWP procedure. Meanwhile, the computational complexity of *AE* can be calculated as $O\left(M\cdot N\right)$. Therefore, the total complexity of the proposed method can be approximated as $O\left(L\cdot M\cdot N\cdot (N-1)+M\cdot N\right)$.

## 5. Experimental Results

In this section, we evaluate the performance of the proposed method discussed in Section 2 for the reconstruction of CDM. We also compare the detection performance of the propose method with that of conventional CD methods.

### 5.1. Description of the Data Sets

**Figure 4:**
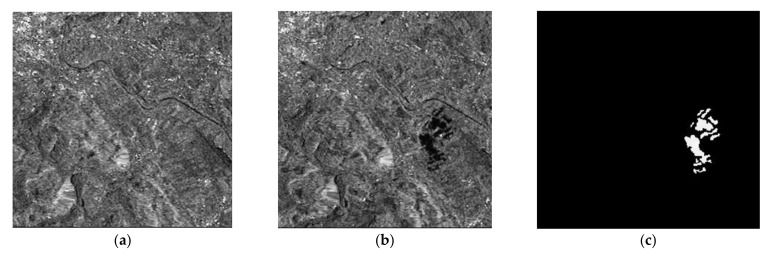
Multi-temporal SAR images of the city of Bern. (**a**) SAR image acquired in April 1999 before the flooding. (**b**) SAR image acquired in May 1999 after flooding. (**c**) The reference map, called ground truth.

**Figure 5:**
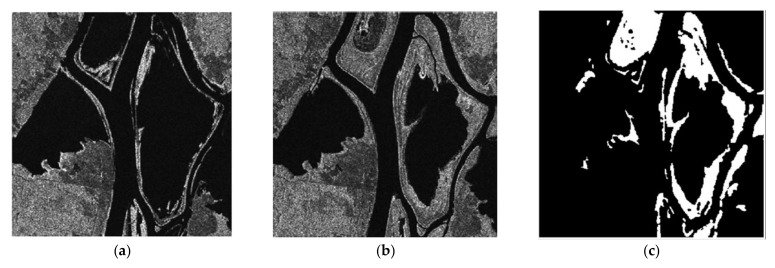
Multi-temporal SAR images of Ottawa. (**a**) SAR image acquired in July 1997 during the flooding. (**b**) SAR image acquired in August 1997 after flooding. (**c**) The reference map, called ground truth.

**Figure 6:**
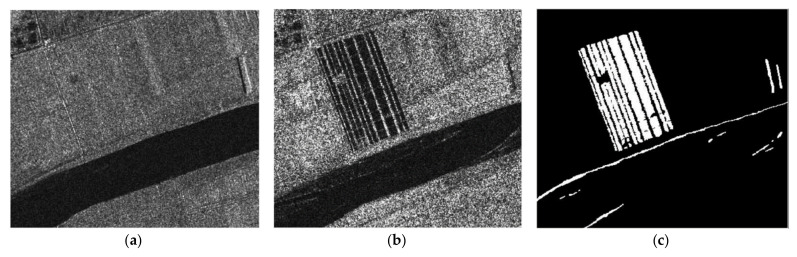
Multi-temporal SAR images of Yellow River Estuary in China. (**a**) SAR image acquired in June 2008. (**b**) SAR image acquired in June 2009. (**c**) The reference map, called ground truth.

To verify the detection performance of the CDM via the proposed method, we used three real SAR dataset (dataset 1, 2 and 3). Before applying the proposed method, it is assumed that the motion compensation algorithm [30] is perfectly performed in advance to generate well-focused SAR images whose phase coherence is well-preserved. Dataset1 consists of two SAR images with a size of 505×494 acquired by the ERS-2 satellite over the same region near the city of Bern, Switzerland, in April and May 1999 as shown in Figure 4a,b, respectively. Dataset2 is comprised of two SAR images with a size of 350×290 obtained from the RADARSAT sensor over the city of Ottawa, Canada, in May and August 1997 as shown in Figure 5a,b, respectively. In both Bern and Ottawa, the specific areas were submerged by floodwater between those two dates, respectively. Dataset3 is pair of two SAR images with a size of 3800×4200 acquired by RADARSAT-2 at the region of Yellow River Estuary in China in June 2008 and June 2009, as shown in Figure 6a,b, respectively. These two images include vegetation areas (paddy field) and are with different levels of strong noise and low coherence. The first image is with four looks and the second image is with a single look. In order to facilitate the quantitative evaluation of creation of CDM for detecting changed areas, reference maps (ground truth images), as shown in Figure 4c, Figure 5c and Figure 6c, were obtained by using manual analysis based on integration of prior information with photo interpretation.

### 5.2. Detection Quality Metrics

We made a direct comparison between each pixel in the result and the reference map to measure the detection quality metrics [13] such as false alarm (*FA*), detection rate (*DR*), and kappa index (κ).

True positive (*TP*) is the number of changed pixels detected correctly and its rate is $p_{TP}=TP/(M\cdot N)$. True negative (*TN*) is the number of unchanged pixels detected correctly and its rate is $p_{TN}=TN/(M\cdot N)$. False positive (*FP*) is the number of changed pixels detected incorrectly as unchanged, also known as misdetections and its rate is $p_{FP}=FP/(M\cdot N)$. False negative (*FN*) is the number of unchanged pixels detected incorrectly as changed pixels and its rate is given as $p_{FN}=FN/(M\cdot N)$. Furthermore, the kappa statistic index, which is a measure of agreement or accuracy based on the difference between the chance agreement and error matrix, was calculated to evaluate the validity and reliability of the CDM, defined as [13]

(10) $$\kappa =\frac{A-B}{1-B},$$

where $A=1-p_{FP}-p_{FN}$ and $B=(p_{TP}+p_{FP})\cdot (p_{TP}+p_{FN})+(p_{TN}+p_{FN})\cdot (p_{TN}+p_{FP})$. The higher the value of kappa, the better is the detection performance. Furthermore, *FA* and *DR* are defined as (FP+FN)/(M⋅N) and TP/CR, where *CR* is the number of pixels as changed region in the reference map, respectively. The final accuracy was evaluated by the proportion of correct detection (*PCD*), which can be calculated by (*TP* + *TN*)/(*TP* + *FP* + *FN* + *TN*).

### 5.3. Analysis of CDMs Generated by the Proposed Method

Under the aforementioned experimental setup, the CC [1], LMT [13], NRA [14], SEST [15], BGD [16], and the proposed method are carried out to compare the detection performance of each CDM. Note that the experiments were conducted to compare the performance of the six CD algorithms in two aspects: (1) before and (2) after post processing. We adopted the median filtering in the post-processing step, which is one of the most popular speckle noise reduction methods, to improve the performance of the CD technique.

**Figure 7:**
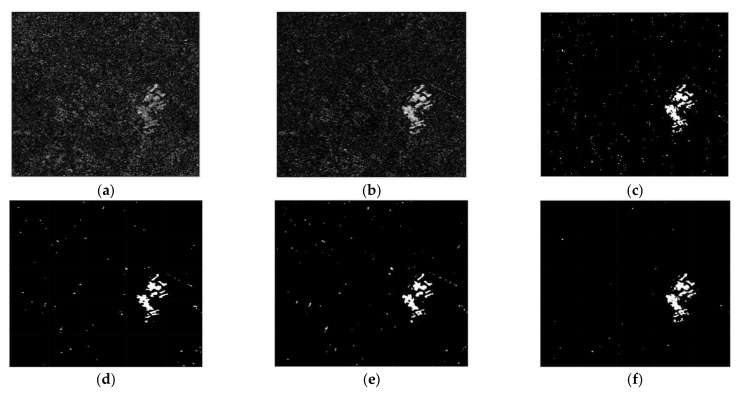
CDMs using dataset 1 obtained by six CD algorithms before median filtering in the post processing step. (**a**) CDM reconstructed by the CC. (**b**) CDM reconstructed by the LMT. (**c**) CDM reconstructed by the NRA. (**d**) CDM reconstructed by the SEST. (**e**) CDM reconstructed by the BGD. (**f**) CDM reconstructed by the proposed method.

**Figure 8:**
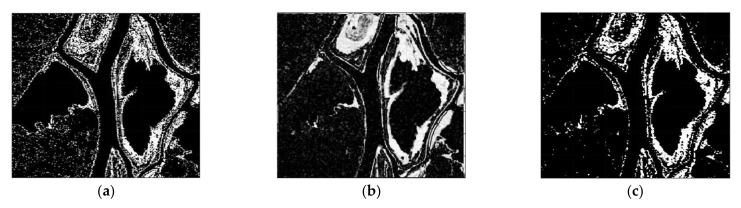

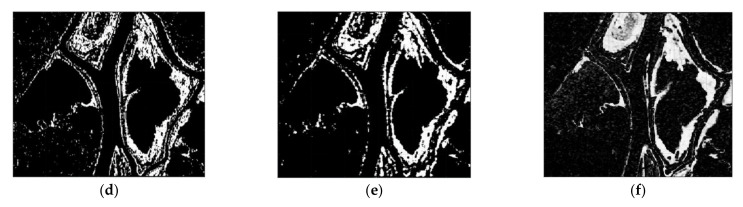
CDMs using dataset 2 obtained by six CD algorithms before median filtering in the post processing step. (**a**) CDM reconstructed by the CC. (**b**) CDM reconstructed by the LMT. (**c**) CDM reconstructed by the NRA. (**d**) CDM reconstructed by the SEST. (**e**) CDM reconstructed by the BGD. (**f**) CDM reconstructed by the proposed method.

**Figure 9:**
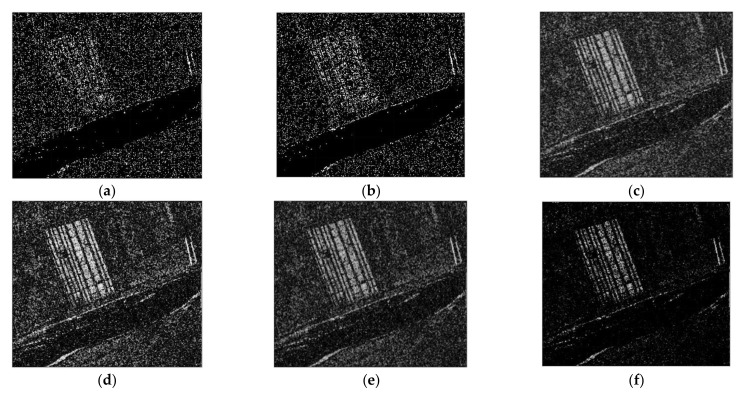
CDMs using dataset 3 obtained by six CD algorithms before median filtering in the post processing step. (**a**) CDM reconstructed by the CC. (**b**) CDM reconstructed by the LMT. (**c**) CDM reconstructed by the NRA. (**d**) CDM reconstructed by the SEST. (**e**) CDM reconstructed by the BGD. (**f**) CDM reconstructed by the proposed method.

**Figure 10:**
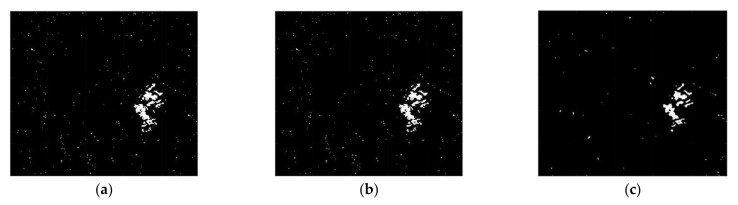

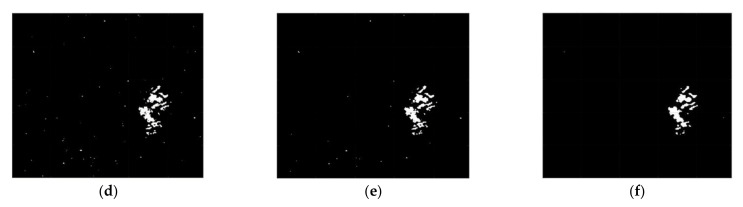
CDMs using dataset 1 obtained by six CD algorithms after median filtering in the post processing step. (**a**) CDM reconstructed by the CC. (**b**) CDM reconstructed by the LMT. (**c**) CDM reconstructed by the NRA. (**d**) CDM reconstructed by the SEST. (**e**) CDM reconstructed by the BGD. (**f**) CDM reconstructed by the proposed method.

**Figure 11:**
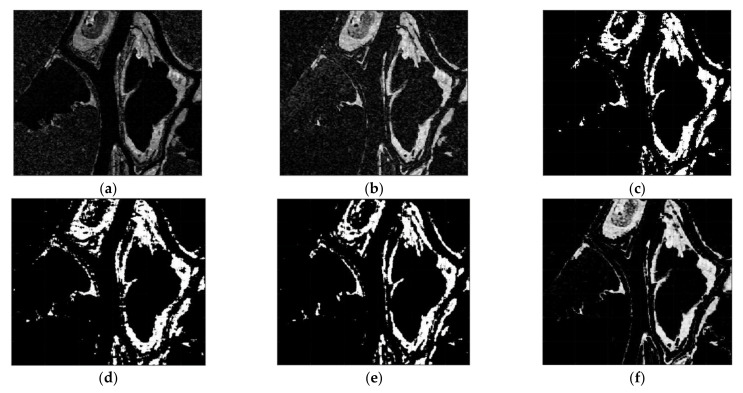
CDMs using dataset 2 obtained by six CD algorithms after median filtering in the post processing step. (**a**) CDM reconstructed by the CC. (**b**) CDM reconstructed by the LMT. (**c**) CDM reconstructed by the NRA. (**d**) CDM reconstructed by the SEST. (**e**) CDM reconstructed by the BGD. (**f**) CDM reconstructed by the proposed method.

**Figure 12:**
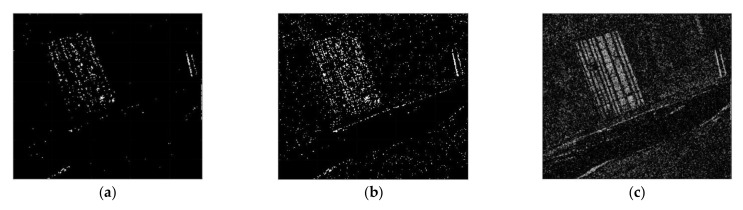

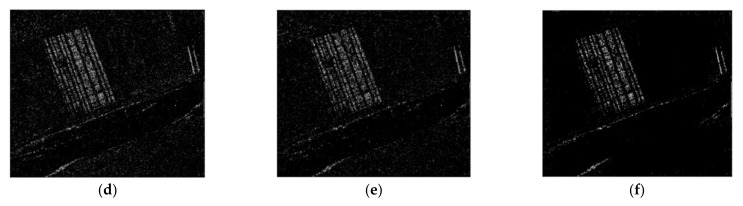
CDMs using dataset 3 obtained by six CD algorithms after median filtering in the post processing step. (**a**) CDM reconstructed by the CC. (**b**) CDM reconstructed by the LMT. (**c**) CDM reconstructed by the NRA. (**d**) CDM reconstructed by the SEST. (**e**) CDM reconstructed by the BGD. (**f**) CDM reconstructed by the proposed method.

The CC, LMT, NRA, SEST, and BGD images are obtained, followed by the application of constant false alarm rate (CFAR) detection to generate the CDMs. Meanwhile, the number of window for the MWP was chosen as *L* = 4 to exploit the preservation of the detail edge of the changed region. Each value of σ for solving the problem of optimization were chosen as −0.002 for dataset 1, −0.015 for dataset 2, and −0.023 for dataset 3, respectively. The main features of the changed regions, such as the flooded areas and the reclaimed paddy fields, are extracted in Figure 7, Figure 8 and Figure 9. It can be easily recognized that the CDM results obtained by the proposed method (depicted in Figure 7f, Figure 8f and Figure 9f) were nearly a match for the reference maps in terms of visual inspection. To provide a quantitative evaluation, the detection performance values of each algorithm are compared in Table 1, Table 2 and Table 3. The *FA* of the CDM obtained by the proposed method has the smallest value among the six approaches in each case (Table 1, Table 2 and Table 3). Furthermore, all the values of kappa and *PCD* measured for the proposed method were considerably greater than those of the other five methods. On the other hand, Figure 10, Figure 11 and Figure 12 show the final CD results obtained by the CC, LMT, NRA, SEST, BGD, and the proposed method, respectively, after the median filtering in the post-processing step. The background of the results obtained using the median filtering is clearer than those of the unfiltered CD results shown in Figure 7, Figure 8 and Figure 9. The detected changed portions in the CD image obtained using the proposed method show good agreement with those of the ground truth map (see Figure 4c, Figure 5c and Figure 6c), as shown in Figure 10f, Figure 11f and Figure 12f. The performances attained by all the methods after the median filtering were described in Table 4, Table 5 and Table 6. The *FA* of the CD image obtained by the proposed method has the smallest value in each dataset among the six algorithms. Furthermore, the *DR* and *PCD* measured by the proposed method are especially better than those of the CC, LMT, NRA, SEST, and BGD. The outstanding performance of the CD image obtained by the proposed algorithm exhibits a significant improvement over other five algorithms in terms of all metrics of detection quality after median filtering in the post processing step. Therefore, the quality of the CDM image obtained by the proposed method was the best in terms of quantitative detection quality measurements in this experiment. In summary, we can conclude that the undesired speckle noises involved in CDMs are successfully suppressed; thus, the CDM quality and detection performance is significantly improved using the proposed method.

## 6. Discussion

The main novelty of the proposed method is two-fold: (1) adoption of the MWP approach based on the SSIM of SAR image with no consideration for the well-preserved interferometric phase, and (2) application of GC in SAR CD framework in order to provide various degrees of performances of both image enhancement and speckle noise reduction with changes in the value of *p*. Because each pixel intensity in the image is closely related to the neighborhood and represents the dynamics of the scene being imaged, the proposed approach can exhibit a simple structure and powerful detection performance. Furthermore, another attractive attribute of the proposed method is that the GC filtering leads to a significant reduction in the speckle noise while retaining nearly all the information related to the true changed region contained in the CDM. In addition, the median filtering can be considered to improve the CD performance in the post processing step. The proposed method is capable of providing a high-quality coherence image since the SSIM operation has high sensitivity to the similarity measure for intensity between two SAR images. Meanwhile, the detection performance of the coherence image is dependent on the order *p* of GC. The proposed method can provide various degrees of performances of the speckle noise reduction with changes in the order *p* of GC. Thus, we analyzed the proposed method in terms of the image enhancement and the speckle noise reduction, taking the value of *p* into consideration. In experimental results, several detection quality metrics are employed to quantitatively evaluate the performance of CDM acquired by the proposed method. The most important issue in the paper is a superior quality of CDM reconstruction by searching the global optimal solutions of the optimization problem with a MWP associated with SSIM and GC, compared with state-of-the-art CD-based methods.

## 7. Conclusions

In this paper, a new framework for NC-CD is proposed for successful CD performance in multi-temporal SAR images. The processing sequence for generation of CDM is implemented as follows: the coherence image is obtained by the MWP associated with the SSIM, followed by optimization procedure of GC. The proposed scheme concentrates on the behavior of the SSIM to measure fine variation of intensity between two SAR images within multiple-windows in order to provide reliable generation of high-quality coherence images. The detection performance of the proposed method is dependent on the order *p* of the power-law function, called GC. The detailed explanation to select the optimum value *p* which plays the lead role in producing the noise-free CDM. The experimental results show that the proposed scheme provides excellent detection performance in terms of several detection quality metrics.

## Figures and Tables

**Table 1:** Comparison of results from all the six methods using dataset 1 before median filtering.

Method	*FP*	*FN*	*FA* (%)	*DR* (%)	κ	*PCD* (%)
CC	601	5918	4.820	67.71	0.164	92.21
LMT	515	5687	3.463	68.22	0.187	93.16
NRA	583	4210	2.966	68.86	0.385	94.45
SEST	479	3786	2.544	69.45	0.534	95.10
BGD	433	3115	2.231	68.02	0.601	94.85
Proposed	378	1643	1.228	65.63	0.794	95.32

**Table 2:** Comparison of results from all the six methods using dataset 2 before median filtering.

Method	*FP*	*FN*	*FA* (%)	*DR* (%)	κ	*PCD* (%)
CC	3263	4015	18.38	69.55	0.677	89.23
LMT	2977	3737	14.64	71.58	0.698	90.10
NRA	2645	3475	11.27	76.79	0.734	91.44
SEST	2712	3615	12.23	78.36	0.749	91.78
BGD	2885	3688	12.72	78.89	0.776	92.21
Proposed	2103	2985	9.668	84.24	0.796	93.43

**Table 3:** Comparison of results from all the six methods using dataset 3 before median filtering.

Method	*FP*	*FN*	*FA* (%)	*DR* (%)	κ	*PCD* (%)
CC	3622	93462	32.69	8.664	0.155	69.74
LMT	3109	80942	24.60	12.57	0.197	75.97
NRA	2279	73947	17.52	16.66	0.288	84.83
SEST	2061	69246	16.41	18.80	0.301	85.45
BGD	1894	67175	15.43	23.78	0.325	86.31
Proposed	1644	63620	14.30	27.44	0.354	87.68

**Table 4:** Comparison of results from all the six methods using dataset 1 after median filtering.

Method	*FP*	*FN*	*FA* (%)	*DR* (%)	κ	*PCD* (%)
CC	564	5491	3.371	70.36	0.204	95.46
LMT	480	5074	2.244	71.15	0.228	97.77
NRA	542	3987	1.830	72.94	0.448	98.12
SEST	503	3451	1.114	73.54	0.635	98.34
BGD	424	2542	0.938	72.27	0.723	98.86
Proposed	319	1359	0.678	68.70	0.848	99.32

**Table 5:** Comparison of results from all the six methods using dataset 2 after median filtering.

Method	*FP*	*FN*	*FA* (%)	*DR* (%)	κ	*PCD* (%)
CC	2771	3523	6.389	74.86	0.735	92.30
LMT	2581	3102	5.670	76.95	0.748	94.43
NRA	2294	2835	5.119	80.10	0.776	94.94
SEST	2018	2621	4.804	82.44	0.782	95.12
BGD	1994	2357	4.676	84.02	0.806	95.46
Proposed	1920	2151	4.062	87.67	0.828	96.01

**Table 6:** Comparison of results from all the six methods using dataset 3 after median filtering.

Method	*FP*	*FN*	*FA* (%)	*DR* (%)	κ	*PCD* (%)
CC	2844	81102	23.20	12.78	0.205	76.41
LMT	2521	74302	18.05	15.55	0.243	80.96
NRA	1897	64482	11.43	19.34	0.318	88.57
SEST	1675	63680	11.21	25.05	0.356	88.89
BGD	1563	60268	10.97	31.31	0.392	89.64
Proposed	1367	58482	10.32	39.53	0.429	90.68

## Data Availability

Not applicable.
